# Effect of Clonidine on Duration of High-Volume, Low-Concentration Caudal Epidurals

**DOI:** 10.7759/cureus.75151

**Published:** 2024-12-05

**Authors:** Christopher Heine, Michelle Rovner, William Irick, Bethany Wolf, Nicole McCoy, Kaylee Massman, Haley Nitchie, Natalie Barnett, Cory Furse

**Affiliations:** 1 Anesthesiology and Perioperative Medicine, Medical University of South Carolina, Charleston, USA

**Keywords:** caudal epidural additives, emergence agitation, pediatric post-operative pain, pediatric regional anesthesia, pediatric urologic surgery

## Abstract

Introduction: Caudal blocks are a common pediatric regional anesthesia technique used to alleviate intra- and postoperative pain following circumcision. The addition of the alpha-2 agonist clonidine has been shown to increase the duration of the block. Another method for prolonging the effect of the block is using a high-volume, low-concentration (HVLC) local anesthetic in the caudal solution. The primary aim of this study was to compare the duration of caudal blockade using HVLC with and without clonidine. The effect of epidural clonidine on postoperative emergence agitation (EA) was assessed as a secondary outcome.

Methodology: This was a prospective, randomized, and observer-blinded study. The participants comprised 129 children, aged 0-3 years, classified as American Society of Anesthesiologists Physical Status Class 1-2, who underwent circumcision and received a caudal injection comprising 1.5 mL/kg of 0.15% ropivacaine and 5 µg/mL of epinephrine after anesthesia induction. The no-clonidine (NC) group received no additional caudal additive, whereas the clonidine (C) group received 1 µg/mL of clonidine. The research team instructed the patients’ parents to administer oral acetaminophen when they detected a pain level corresponding to a visual analog scale score ≥4 on the Wong-Baker FACES Pain Rating Scale or when they felt that their child was uncomfortable. EA occurrence was measured by post-anesthesia care unit nurses using the Pediatric Anesthesia Emergence Delirium (PAED) scale.

Results: No significant differences were observed between the two groups regarding demographics, anesthesia, surgery, or discharge time. The median time to the first postoperative acetaminophen dose was 335 min for the NC group and 381 min for the C group (P = 0.901). The NC group had a mean PAED score of 6.5 compared with 6.4 in the C group (P = 0.894).

Conclusion: In children who underwent circumcision, adding clonidine to the HVLC caudal injection of ropivacaine did not prolong the time to the first acetaminophen dose or the occurrence of EA, suggesting no benefit in using clonidine in this surgical population.

## Introduction

Caudal blocks are commonly performed by anesthesiologists during pediatric abdominal or lower-extremity surgeries because of their effectiveness, ease of placement, and low complication rates [1]. These caudal blocks are often administered at the beginning of an operation to provide intra- and postoperative pain control and minimize the supplemental analgesia requirement. The duration of longer-acting local anesthetics such as ropivacaine and bupivacaine has led to their widespread use for regional anesthesia. Caudal blocks administered with plain bupivacaine and ropivacaine result in long-lasting pain relief for up to 2-12 h, depending on their concentration and volume [2,3].

The calculated dose of the local anesthetic delivered combines volume and concentration. The larger the volume administered, the greater the cranial spread of the local anesthetic within the caudal space, and the greater the number of dermatomes with an analgesic effect. However, a higher concentration leads to a denser sensory block and possibly motor blockade. Multiple formulas have been used to determine the optimal volume for an appropriate blockade level [4]; a common formula of 1 mL/kg up to a maximum volume of 20 mL is commonly used to achieve a T-10 dermatome sensory block. Varying volumes and concentrations have been studied to determine the amount that maximizes benefits while minimizing risks and side effects [5-8].

Caudal duration and density may be altered in two ways. Additives such as epinephrine, dexamethasone, ketamine, opioids, neostigmine, and alpha-2 agonists such as dexmedetomidine and clonidine can increase the block effect. Epinephrine is often used not only to help improve the block but also to warn the practitioner of an unintended systemic injection with resulting tachycardia or T-wave changes on an electrocardiogram. Other additives such as clonidine are used primarily for their effects on the characteristics of the block; 1-2 µg/kg of clonidine can significantly increase the block duration [9-11].

The second way to change the duration is by adjusting the volume or concentration of the local anesthetic. Larger volumes lead to greater coverage of the block area and higher concentrations lead to denser blocks. Combining a higher volume of injectate with a lower but still efficacious concentration can significantly increase analgesia duration from caudal blocks while also preventing side effects from higher concentrations of the local anesthetic. This technique has been termed “high volume/low concentration” (HVLC). When compared to a “low volume/high concentration” (LVHC) injection of 1.0 mL/kg of 0.225% ropivacaine, an HVLC of 1.5 mL/kg of 0.15% ropivacaine (note, same total dose of local anesthetic of 2.25 mg/kg) results in increased time to the first acetaminophen rescue dose [5].

The addition of clonidine to an HVLC caudal injection has not yet been studied. The use of clonidine may increase the analgesia duration without additional side effects. Alpha-2 agonists, such as clonidine, administered in the caudal space have also been associated with a decreased incidence of emergence agitation (EA) in the recovery room. One study that compared the use of intravenous and caudally administered clonidine and its effects on EA found that clonidine had a dose-dependent effect on lowering EA incidence and its efficacy was unaffected by the route of administration [12].

In this study, we compared the primary outcome of caudal blockade duration in HVLC ropivacaine with and without clonidine. EA was a secondary outcome, and hemodynamic changes, sedation levels in the recovery room, and time to discharge were also reported.

## Materials and methods

Participants

This study was conducted by the Department of Anesthesia and Perioperative Services at the Medical University of South Carolina (MUSC) in Charleston, South Carolina, USA. It was approved by the MUSC Institutional Review Board in 2020. The study was registered with ClinicalTrials.gov prior to enrollment (Identifier NCT04263064; registration date: February 10, 2020). This prospective, randomized, observer-blinded trial was conducted between March 2020 and August 2022.

Patients who presented for outpatient circumcision were included if they were aged ≤3 years, weighed <13.3 kg, classified as American Society of Anesthesiologists Physical Status Class 1 or 2, and the anesthetic plan included a caudal block. Patients were excluded if they had a known allergy to clonidine, epinephrine, or amide local anesthetics or if a parent was unable or unwilling to provide informed consent.

Eligible patients were identified by the study team one week prior to their surgical date and the study information was mailed to their parents. A formal description of the study was provided to the parents and written informed consent was obtained by the study team on the day of surgery. Parents were instructed on how to use the visual analog scale (VAS) and interpret the Wong-Baker FACES Pain Rating Scale score (0 = no pain, 10 = worst pain possible) before discharge. A total of 129 patients were enrolled in this study.

Anesthesia

The anesthesiologist who performed the anesthetic procedure, including the caudal block, was not blinded. The participating anesthesiologists were aware of the outcome goals of the study but were instructed to administer the anesthetic that they determined was most appropriate for the patient. Premedication, parental presence for anesthesia induction, induction technique, anesthesia maintenance, analgesia, and emergence technique were decided at the discretion of the anesthesiologist and were recorded by a blinded observer.

The standard anesthetic plan for this patient population in our institution is mask anesthesia induction with 8% sevoflurane with or without 70% nitrous oxide, laryngeal mask airway (LMA) placement after obtaining IV access, caudal epidural administration, maintenance with sevoflurane, deep LMA removal before leaving the operating room, and admission to the post-anesthesia care unit (PACU) for observation.

Intervention

The participants were assigned to one of two groups: HVLC without clonidine (NC) (1.5 mL/kg of 0.15% ropivacaine and 5 µg/mL epinephrine) and HVLC with clonidine (C) (1.5 mL/kg of 0.15% ropivacaine with 1 µg/mL of clonidine and 5 µg/mL epinephrine). The operating room pharmacist followed the randomization and prepared the assigned caudal solutions. The observer who recorded the patient data and the recovery room nurses were blinded to the study group.

All caudal blocks were performed with the patient in the lateral position using a sterile technique. The sacral cornu and ligaments were identified using landmark palpation. The caudal space was accessed using a 22-gauge short-bevel needle and a 1 mL test dose was administered. Following a negative test result, the study drugs were administered incrementally and the patient was placed supine for the procedure. Surgery was not allowed to start until 10 min after drug administration to provide sufficient time for the caudal injection to take effect.

Assessments

After the procedure was completed and the patient arrived in the PACU, the PACU nurse monitored, evaluated, and managed the patient according to the standard care. Rescue analgesia was administered at the prescribed dose if two successive observations yielded a Children’s Hospital of Eastern Ontario Pain Scale (CHEOPS) score ≥4. Postoperative sedation was evaluated using the 8-point modified Ramsey Sedation Scale. EA occurrence was evaluated using the Pediatric Anesthesia Emergence Delirium (PAED) scale and the need for rescue medication was recorded. These scores were recorded every 15 min until discharge, the readiness for which was defined as an Aldrete score ≥9.

The observer recorded the patient demographics prior to anesthesia induction. Intraoperatively, they recorded the use of premedication or parental presence for anesthesia induction, procedure and anesthesia duration, intravenous fluid type and volume, use of intraoperative analgesics, and the depth of anesthesia with airway removal. In the PACU, the observer recorded the use of rescue pain medications, recovery time, and pain and EA scores calculated by the nurses.

The parents were instructed by the research team to administer oral acetaminophen when they detected a pain level corresponding to a VAS score of ≥4 on the Wong-Baker FACES Pain Rating Scale, or if they felt that their child was uncomfortable. Approximately 24 h after the surgery, a phone interview with the parent was conducted by the observer and the time of first acetaminophen administration was recorded.

Statistical analyses

The difference between block durations by block type was evaluated using a two-sample t-test approach or a Wilcoxon rank-sum test if the normality assumption did not hold. Hong et al. [5] found that children undergoing pediatric orchiopexy who received an HVLC ropivacaine caudal block without clonidine had a median block duration of approximately 555 min (interquartile range [IQR] = 370, which yielded an estimated standard deviation of IQR/1.4 = 260).

An increase in block duration by 2.5 h (150 min) from 555 min to 705 min was considered clinically meaningful. A sample size of 50 participants per group provided an 80% power to detect a 2.5 h increase in block duration, assuming that the mean block duration in the patients who received caudal blocks without clonidine was 555 ± 260 min at a significance level α = 0.05 using a two-sided Wilcoxon rank-sum test.

Descriptive statistics by treatment arm were calculated for all the patients and their procedural characteristics. The primary outcome of interest was time to the first acetaminophen dose. Approximately 14% of the participants either did not receive acetaminophen or were lost to follow-up and were thus right-censored for time to analgesia. To account for censoring, differences in the time to the first acetaminophen dose by treatment group were examined using a univariate Cox regression analysis. The participants who were lost to follow-up were censored at the time of hospital discharge, whereas those who did not receive acetaminophen were censored at the maximum time a participant received acetaminophen. The proportional hazards assumption was verified using the Grambsch-Therneau test.

Secondary outcomes included postoperative sedation using the Ramsey scale, pain determined by the CHEOPS, and EA by the PAED scale measured over time postoperatively. Differences in the scores between the treatment groups for each tool during the postoperative period were evaluated using a series of linear mixed models. The models included fixed effects for the treatment group and postoperative time and a random participant effect to account for the correlation of measures collected for the same patient over time. The original study evaluated these measures at 0, 30, 60, and 90 min postoperatively. However, in our study, since most of our patients arrive in the recovery room in a deep plane of anesthesia, only eight children qualified for EA evaluation at 0 min. Furthermore, only 20 children had not met PACU recovery criteria and were, therefore, present at 60 min for evaluation, and all had been discharged by 90 min; therefore, the interaction between the treatment group and postoperative time was not considered in the model. All model assumptions were checked graphically, and transformations were considered if necessary.

All analyses were conducted using SAS v. 9.4 (SAS Institute, Cary, NC).

## Results

The final study population included 125 participants, 65 in the NC group and 60 in the C group. Participant and procedural characteristics for each treatment arm are presented in Table 1. Approximately half of the study population were White, 34.4% were Black, and 17.6% were of other or unknown races. The mean patient age was 17 ± 9 months. No notable differences in patient or procedural characteristics were observed between the treatment groups. 

**Table 1:** Patient and procedural characteristics by treatment group BMI, body mass index; HVLC, high volume/low concentration; SD, standard deviation; IQR, interquartile range; MAC, minimum alveolar concentration.

Patient and procedural characteristics	HVLC (N = 65)	HVLC + clonidine (N = 60)	P-value
Age: months, mean (SD)	17.4 (8.92)	17.6 (9.28)	0.918
Race, n (%)			0.104
White	27 (41.5)	33 (55.0)	
Black	28 (43.1)	15 (25.0)	
Other/Unknown	10 (15.4)	12 (20.0)	
Height: cm, mean (SD)	75.1 (6.39)	74.6 (6.97)	0.717
Weight: kg, mean (SD)	10.0 (1.54)	10.1 (1.47)	0.925
BMI: kg/m^2^, mean (SD)	17.8 (2.28)	18.3 (3.76)	0.425
Premedication required: Yes, n (%)	8 (12.3)	14 (23.3)	0.106
Level of anesthesia at airway removal, n (%)			0.681
Awake	4 (6.25)	2 (3.33)	
Deep	60 (93.8)	58 (96.7)	
Duration of anesthesia: min, mean (SD)	41.8 (6.76)	41.7 (6.04)	0.740
Procedure duration: min, mean (SD)	19.9 (4.34)	20.1 (3.93)	0.819
Fluid volume: mL, median (IQR; range)	200 (140; 0, 350)	200 (100; 0, 400)	0.897
Total postoperative time: min, mean (SD)	47.5 (11.8)	52.5 (25.9)	0.764
Maintenance MAC: median (IQR; range)	1.12 (0.2; 0.7-3.6)	1.09 (0.2; 0.78-3.1)	0.380

Time to the first acetaminophen dose

**Figure 1:**
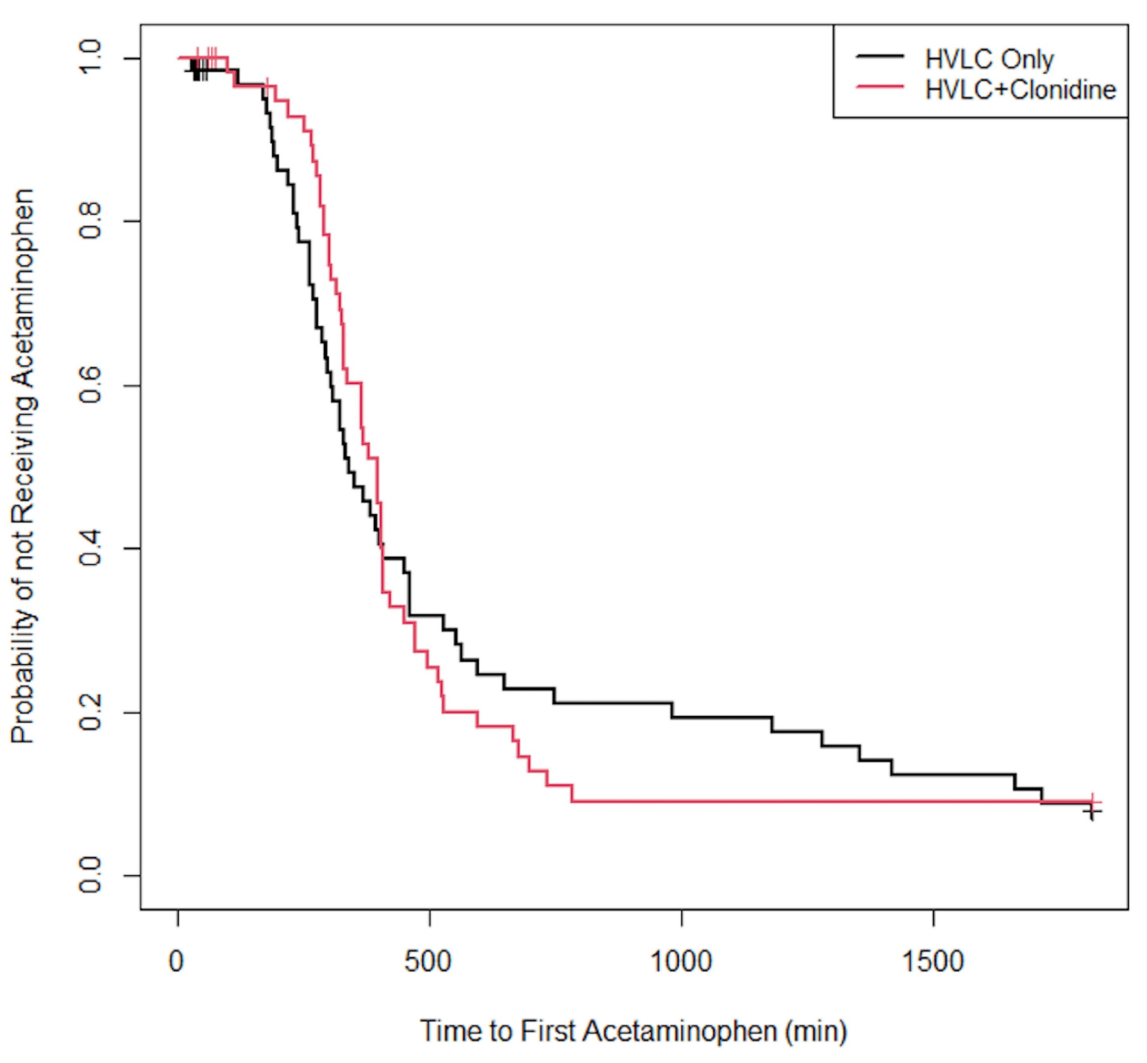
Kaplan-Meier curves for time to the first acetaminophen dose by treatment group HVLC, high volume/low concentration.

Approximately 82% of the participants were reported as having received acetaminophen at home. Specifically, among the patients in the NC group, 53 received acetaminophen at home, four did not receive acetaminophen, and eight were lost to follow-up. Among the patients in the C group, 50 received acetaminophen at home, five did not receive acetaminophen, and five were lost to follow-up. The median time to the first acetaminophen dose in the NC group was 335 min compared to 381 min in the C group (P = 0.901). The Kaplan-Meier curves for the probability of not having received acetaminophen are displayed in Figure 1.

Postoperative sedation, pain, and EA

No notable differences in postoperative sedation, pain, or EA were observed between the two groups. The average sedation level for the NC group was 2.1 compared with 2.2 for the C group (P = 0.670). The NC group had a mean CHEOPS score of 3.2 compared with 2.9 for the C group (P = 0.424). The NC group had a mean PAED score of 6.5 compared with 6.4 for the C group (P = 0.894). All treatment group outcomes are presented in Table 2.

**Table 2:** Study outcomes by treatment group HVLC, high volume/low concentration; CHEOPS, Children’s Hospital of Eastern Ontario Pain Scale; PAED, Pediatric Anesthesia Emergence Delirium. *Participants not reporting “Yes” were those who reported not giving the child any acetaminophen and children lost to follow-up. ^+^Median time to the first acetaminophen administration and 95% confidence intervals were estimated using the Brookmeyer–Crawley approach with log-log complement transformation. The P-value was obtained from the univariate Cox regression model for the time to the first acetaminophen dose. ^Ϯ^Mean and 95% confidence intervals were estimated from linear mixed models, including fixed effects for the treatment group, postoperative time, and random participant effect.

Outcome	HVLC only (N = 65)	HVLC + clonidine (N = 60)	P-value
Acetaminophen administered at home: Yes, n (%)*	53 (81.5)	50 (83.3)	0.819
Time to acetaminophen: min, median (95% CI)^+^	335 (295, 408)	381 (330, 408)	0.902
Ramsey score: mean (95% CI)^Ϯ^	2.1 (1.9, 2.4)	2.2 (1.9, 2.5)	0.670
CHEOPS score: mean (95% CI)^Ϯ^	3.2 (2.5, 3.8)	2.9 (2.2, 3.6)	0.424
PAED score: mean (95% CI)^Ϯ^	6.5 (5.1, 7.8)	6.4 (5.0, 7.8)	0.894

## Discussion

The addition of clonidine to the HVLC caudal preparation resulted in no significant differences in the time to the first dose of acetaminophen or the administration rate in the first 24 h postoperatively. Additionally, no significant differences were observed in the secondary outcomes for sedation levels or EA noted in the PACU, as measured by the Ramsey and PAED scores, respectively. Although the use of clonidine as an adjuvant did not appear to result in any negative effects as measured in this study, it did not lead to any beneficial effects.

Our primary outcome, the time to acetaminophen administration, is likely the most reliable measure of the duration of an effective caudal block in this study population. Conducting this study with a surgical population that could report their own pain scores, admitted overnight, and assessed by nurses with extensive experience in pain assessment may have influenced the outcomes. However, this approach was not feasible for the current study. Caudal injections are rarely administered to children who are old enough to self-report pain, and our surgical volumes for procedures requiring overnight admission would have made it difficult to enroll a sufficient number of patients undergoing the same surgery.

The use of clonidine in the caudal space is not without risk [13]. Although largely considered safe at the dose used in this study, there are case reports of postoperative respiratory depression and apnea in premature infants attributed to the addition of clonidine in caudal blocks [14,15]. Intravenous clonidine has been shown to blunt the respiratory response to carbon dioxide [16]. This response is further altered by many drugs commonly administered as part of general anesthesia. This response raises concern, particularly for outpatient procedures in patients born prematurely. Premature infants and neonates who receive clonidine as an additive in the caudal region should be observed postoperatively, according to their respective institutional guidelines, regardless of whether they also receive a general anesthetic.

Clonidine is used as an antihypertensive medication in some patients owing to its central alpha-2 receptor-activating property. This mechanism of action has also been associated with bradycardia and hypotension when clonidine is used as an additive in epidural infusions, although this is more commonly observed in adults [17,18]. When used as an intravenously administered sedative, it has been shown to result in bradycardia and hypotension in critically ill children [19]. Since the cardiac output of neonates and young infants is dependent on heart rate due to an immature myocardium, clonidine should be used with caution in this population [20]. 

This study had limitations. First, obtaining objective pain scores in young, non-verbal children is challenging, and we relied on a brief discussion with parents on how to utilize the pain scales. This may have led parents to administer acetaminophen earlier than intended, which could explain the relatively short duration of blockade observed in both groups. Educating parents on the use of pain scores to determine when to administer an analgesic was employed in a previous study with a similar design. However, Hong et al. [5] reported a significant increase in the duration of caudal epidural anesthesia with the use of an HVLC solution compared with the use of an LVHC solution (554 vs. 363 min, P < 0.001). Additionally, we instructed parents to administer acetaminophen if they felt that their child was uncomfortable. This subjective measurement might explain why both of our groups had similar times to the first dose of acetaminophen as the LVHC group in the Hong study.

## Conclusions

Our results suggest that adding clonidine to an HVLC solution of ropivacaine does not extend the time to the first postoperative administration of acetaminophen in children who undergo circumcision. This approach does not seem to reduce the risk of EA in these patients. Given that clonidine has well-documented side effects, using it in caudal epidurals should be done with caution.
